# Impact of Use Frequency of a Mobile Diabetes Management App on Blood Glucose Control: Evaluation Study

**DOI:** 10.2196/11933

**Published:** 2019-03-07

**Authors:** Josep Vehi, Jordi Regincós Isern, Adrià Parcerisas, Remei Calm, Ivan Contreras

**Affiliations:** 1 Institut d'Informatica i Aplicacions Universitat de Girona Girona Spain; 2 Centro de Investigación Biomédica en Red de Diabetes y Enfermedades Metabólicas Asociadas Girona Spain

**Keywords:** diabetes mellitus, mHealth, self-management, blood glucose self-monitoring, evaluation studies

## Abstract

**Background:**

Technology has long been used to carry out self-management as well as to improve adherence to treatment in people with diabetes. However, most technology-based apps do not meet the basic requirements for engaging patients.

**Objective:**

This study aimed to evaluate the effect of use frequency of a diabetes management app on glycemic control.

**Methods:**

Overall, 2 analyses were performed. The first consisted of an examination of the reduction of blood glucose (BG) mean, using a randomly selected group of 211 users of the SocialDiabetes app (SDA). BG levels at baseline, month 3, and month 6 were calculated using the intercept of a regression model based on data from months 1, 4, and 7, respectively. In the second analysis, the impact of low and high BG risk was examined. A total of 2692 users logging SDA ≥5 days/month for ≥6 months were analyzed. The highest quartile regarding low blood glucose index (LBGI) and high blood glucose index (HBGI) at baseline (t1) was selected (n=74 for group A; n=440 for group B). Changes in HBGI and LBGI at month 6 (t2) were analyzed.

**Results:**

For analysis 1, baseline BG results for type 1 diabetes mellitus (T1DM) groups A and B were 213.61 (SD 31.57) mg/dL and 206.43 (SD 18.65) mg/dL, respectively, which decreased at month 6 to 175.15 (SD 37.88) mg/dL and 180.6 (SD 40.47) mg/dL, respectively. For type 2 diabetes mellitus (T2DM), baseline BG was 218.77 (SD 40.18) mg/dL and 232.55 (SD 46.78) mg/dL, respectively, which decreased at month 6 to 160.51 (SD 39.32) mg/dL and 173.14 (SD 52.81) mg/dL for groups A and B, respectively. This represents a reduction of estimated A_1c_ (eA_1c_) of approximately 1.3% (*P*<.001) and 0.9% (*P*=.001) for T1DM groups A and B, respectively, and 2% (*P*<.001) for both A and B T2DM groups, respectively. For analysis 2, T1DM baseline LBGI values for groups A and B were 5.2 (SD 3.9) and 4.4 (SD 2.3), respectively, which decreased at t2 to 3.4 (SD 3.3) and 3.4 (SD 1.9), respectively; this was a reduction of 34.6% (*P*=.005) and 22.7% (*P*=.02), respectively. Baseline HBGI values for groups A and B were 12.6 (SD 4.3) and 10.6 (SD 4.03), respectively, which decreased at t2 to 9.0 (SD 6.5) and 8.6 (SD 4.7), respectively; this was a reduction of 30% (*P*=.001) and 22% (*P*=.003), respectively.

**Conclusions:**

A significant reduction in BG was found in all groups, independent of the use frequency of the app. Better outcomes were found for T2DM patients. A significant reduction in LBGI and HBGI was found in all groups, regardless of the use frequency of the app. LBGI and HBGI indices of both groups tend to have similar values after 6 months of app use.

## Introduction

### Background

Diabetes mellitus (DM) is a chronic disease with a major impact on morbidity and mortality as well as socioeconomics [1]. This impact is because of its high prevalence and incidence as well as the associated acute and chronic complications, which are caused by poor glucose control [1]. Therefore, self-management of blood glucose (BG) is the standard of care for people with diabetes [2]. On the basis of previous studies, it has been found that for those with type 2 diabetes (T2DM) BG can be maintained through physical activity, healthy diet, and weight loss [3-5]. For people with type 1 diabetes (T1DM), it is more difficult to control through diet and exercise. Despite this, current studies suggest that those with T1DM and T2DM do not adhere to these recommendations [4,6]. Mobile health (mHealth) apps could be a solution to promote adherence to physical activity and weight reduction regimens. However, current apps require intensive one-on-one or group lifestyle coaching [7].

Technology has long been used in self-management and to improve treatment adherence in people with diabetes [8-10]. Systems based on telephone coaching, short message service support, or telemedicine have proven effective in increasing management adherence and, consequently, improving glycemic control [9,10]. Currently, the global implementation of mobile phones has fostered the development of apps for diabetes management, which have become primary tools for decision support and disease management for both people with diabetes and health care providers [11]. However, some of these apps have not been proven to work in real life, and some studies have observed that they generally do not meet the basic requirements for engaging the patient [12-14].

Retrospective studies of diabetes management apps have recently been reported, which have demonstrated a reduction in mean glucose [15-18], glycated hemoglobin (HbA_1c_) levels, and the risk of hypoglycemia for patients who used an app frequently [18-20]. However, many of these studies have focused on the improvement of BG control for adherent patients rather than on the level of adherence needed to obtain this impact on glycemic control [21,22]. For this reason, other recent reviews have demonstrated that very few of these apps use this information to provide users with personalized feedback, education, or motivation [23,24].

Thus, starting from the premise that an app that promotes patient education toward their disease and assists in increasing the efficacy of their treatment and self-regulation is needed, we have performed an analysis on a current mHealth app to devise a methodology to make apps more effective for this purpose. The primary objective of this study was to evaluate the effect of the use frequency of a diabetes management app on glycemic control in participants with DM. The results presented in this paper correspond to 2 retrospective analyses. The first analysis consisted of examining the effect of the use frequency of a diabetes management app on the reduction of estimated HbA_1c_ levels. The goal of the second study was to examine the app’s impact on the low blood glucose index (LBGI) and high blood glucose index (HBGI).

### SocialDiabetes System

**Figure 1:**
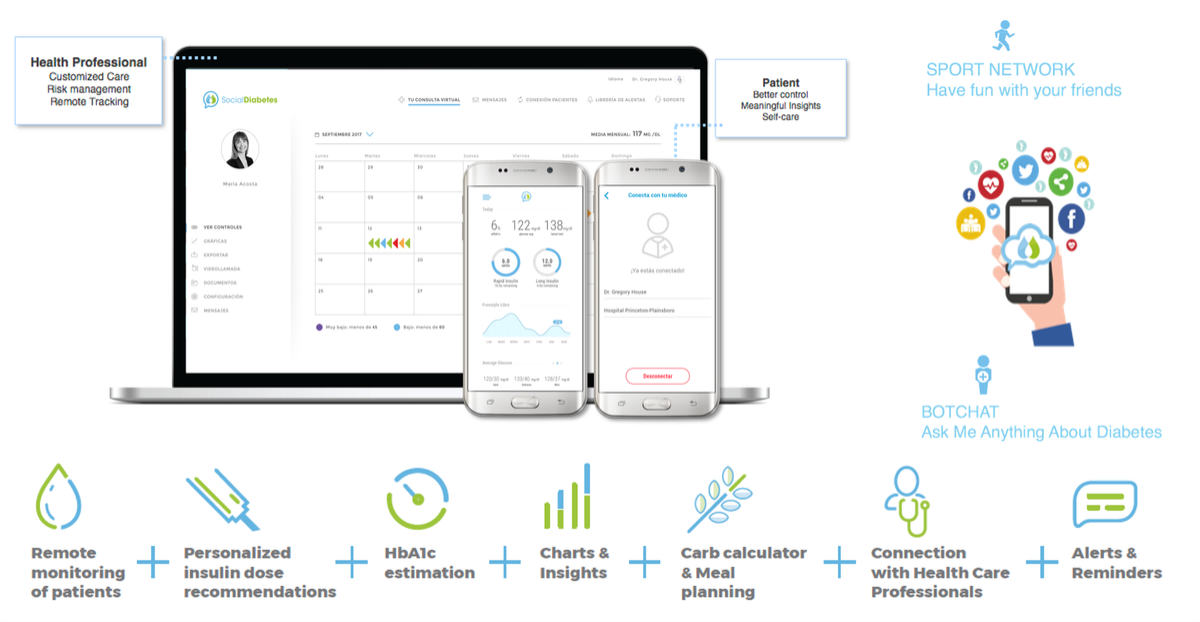
General scheme of the SocialDiabetes integrated platform.

SocialDiabetes is an independent digital health care platform for diabetes management, created by people with diabetes to transform the everyday life of patients by unlocking the potential of data-driven innovation and community development. The platform is complete with a mobile app and a desktop solution that empowers diabetes patients to actively engage in their own care. A global vision of the platform and its characteristics are shown in Figure 1.

Using the SocialDiabetes app (SDA), patients can sync their BG data from their meter to their phone and add any other relevant information in real time (ie, exercise, food, and lifestyle). Professional practitioner care teams employed by the app are connected to the patients through the SDA care Web platform and can remotely monitor and track their progress. Data are accessible to both patients and health care providers. In the near future, SDA will incorporate a community platform. Its intention is to bring the possibility of facilitating communication not only between the patient and physician but also between all patients with diabetes with similar characteristics. In this manner, each patient will be able to explain their experience to others.

Some basic tools such as remote monitoring of patients, HbA_1c_ estimation, carbohydrates calculators, alerts, and reminders characterize the platform. As they encourage patient personalization, the app also provides personalized insulin dose recommendations, connection with health care professionals, meal planning, exercise coaching and charts, and insights about the parameters and statistics of every patient.

The objective of this study was to demonstrate by means of 2 analyses on 2 independent indicators of glycemic control that the impact of a diabetes management app on glycemic control is more related to its monthly users’ usage in a consistent manner rather than daily, weekly or monthly high-frequency use. When we talk about consistency, we are referring to a habit that is followed on a regular basis and over a long period. So, the use of the app is a part of his/her diabetes management routine. In other words, the frequency of use necessary to generate consistency is a specific parameter for each individual. Therefore, the frequency of use is not a determinant for glycemic control as long as it is sufficient to generate a consistent use of the app. Thus, users logging into the app on a monthly basis for a long time can have an impact on glycemic control regardless of whether their frequency of use is high or low.

## Methods

### Description of Study Participants

In this study, 2 different measures for glycemic control have been conducted: the first evaluating the HbA_1c_ levels through the frequency of use of the app and the second examining its impact on LBGI and HBGI.

To perform the study, a subset of informed consenting users of the SDA database was first selected. The inclusion criteria were 18 years old or more, more than 1 year with diabetes, and no complications. An additional engagement inclusion criterion was applied, which consisted of an analysis of an app usage report where users who were logging in at least 5 days per month during at least 6 months (logging ≥5 days/month for ≥6 months) were selected. Users have been previously distinguished according to T1DM and T2DM as disease management between the two is often very different. Finally, to have an adequate sample of users and to obtain more accurate results, a different inclusion/exclusion criterion was used for each of the 2 analyses.

### Reduction of Estimated Glycated Hemoglobin

A total of 211 users of the SDA were included in the analysis, among which 144 (68.2%) participants had T1DM and 67 (31.8%) had T2DM. For this analysis, a mean BG inclusion criterion was applied, where participants with mean BG levels at baseline more than or equal to 183 mg/dL (representing eA_1c_ ≥8%) were selected.

Among the users, 19 (9%) had a body mass index (BMI) less than 18.5, 98 (46.4%) had between 18.5 and 25, 74 (35.1%) had between 25 and 30, and 20 (9.5%) had greater than 30. Almost half of the selected users (99/211) were diagnosed between 10 and 30 years ago, 40.3% (85/211) was diagnosed less than 10 years ago, and the other users (27/211) were diagnosed over 30 years ago.

In addition, 144 (68.2%) users had T1DM, of which 57.6% (83/144) were male and 42.4% (61/144) were female. The mean age of males was statistically higher than that of females, 34.8 (SD 4.22) years versus 31.93 (SD 3.14) years, respectively. Conversely, participants with T2DM constituted 31.8% (67/211) of the total participants, with 74.6% (50/67) being male and 25.4% (17/67) being female. In this case, the mean age of males was higher than that of females, 53.2 (SD 4.02) years for males and 49.1 (SD 4.11) years for females.

The cohort was split into 2 groups according to the intensity of app engagement: group A represents the high-engagement group (logging ≥15 times/month for ≥6 months) and group B represents the low-engagement group (logging 5-10 times/month for ≥6 months). Applying this criterion, group A represents 57% (82/144) of T1DM participants and 67% (45/67) of participants with T2DM. The other participants for both T1DM and T2DM were included in group B, representing 43% (62/133) of T1DM and 33% (22/45) of T2DM participants.

BG levels at baseline, month 3, and month 6 were calculated using the intercept of a regression model based on data from months 1, 4, and 7, respectively. Estimated HbA_1c_ was calculated at baseline, month 3, and month 6 using linear regression analysis as described in the study by Holmes et al [22]. Paired *t* test was used to test the mean difference between baseline, month 3, and month 6.

### Reduction of Risks of Hypoglycemia and Hyperglycemia

A total of 2692 users of the SDA were analyzed, of which 2248 (83.5%) of the participants had T1DM and 444 (16.5%) of the participants had T2DM. The inclusion criterion of this analysis consisted of engagement.

Among the subset selected, 161 (6%) participants had a BMI less than 18.5, 1146 (42.6%) had between 18.5 and 25, 779 (28.9%) had between 25 and 30, and 606 (22.5%) had greater than 30. More than half of them were diagnosed less than 10 years ago (1404/2692), 37.5% (1010/2692) were diagnosed between 10 and 30 years ago, and the other users (278/2692) were diagnosed over 30 years ago.

Of the participants, 2248 (83.5%) had T1DM, of whom 59% (1326/2248) were male and 41% (922/2248) were female. The mean age for the males was statistically higher than that of the females, 36.7 (SD 4.98) years versus 31.81 (SD 3.34) years, respectively. Conversely, participants with T2DM constituted 16.5% (444/2692) of the total participants, with 79.3% (352/444) being male and 20.7% (92/444) being female. In this case, the mean age of males was higher than that of females, 55.2 (SD 4.22) years for male and 48.7 (SD 4.41) years for females.

The cohort was split into 2 groups according to the intensity of app engagement: group A, the high-engagement group (logging ≥60 times/month for ≥6 months), and group B, the low-engagement group (logging 5-10 times/month for ≥6 months). Applying this criterion, group A represents 86% of T1DM participants (1944/2248) and 66% of participants with T2DM (292/444). The other participants for both T1DM and T2DM were included in group B, representing 14% (304/2248) of T1DM and 34% (152/444) of T2DM participants.

The LBGI and HBGI were calculated according to the methods described in the study by Nathan et al [25]. It is well known that these indexes correlate with the risk of having hypoglycemia and hyperglycemic events, respectively [26].

From each group, the highest quartile regarding LBGI and HBGI at baseline (t1) was selected (n_1_=486 and n_2_=73 for group A; n_1_=76 and n_2_=38 for group B, where n_1_ and n_2_ refers to the number of T1DM and T2DM users respectively). Changes in HBGI and LBGI at month 6 (t2) were analyzed. Paired *t* test was used to compare HBGI and LBGI at baseline with those at month 6.

## Results

### Reduction of Estimated Glycated Hemoglobin

**Figure 2:**
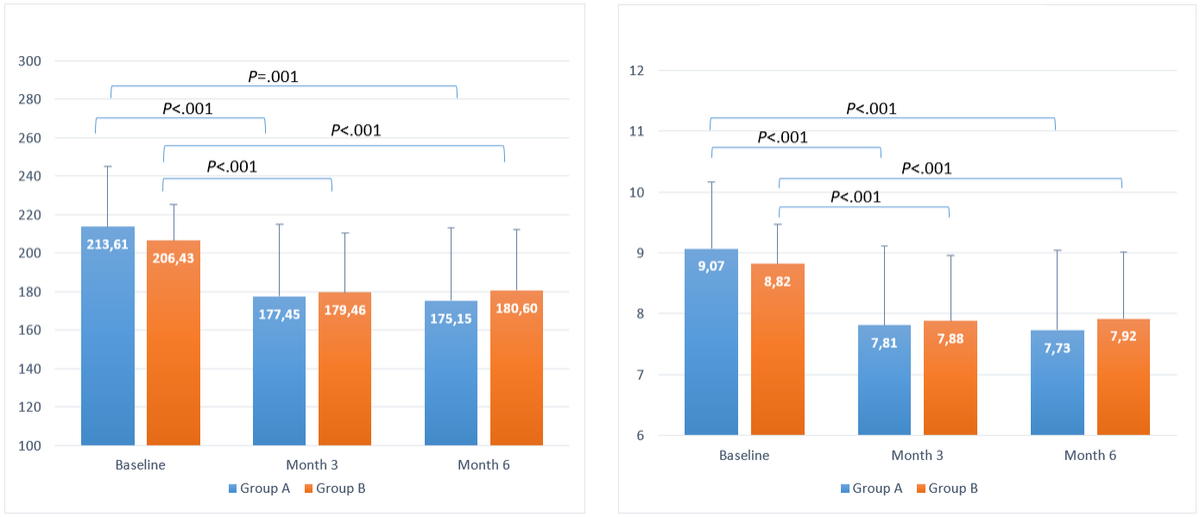
Timeline evolution of estimated blood glucose (left) and estimated glycated hemoglobin (right) for users with type 1 diabetes mellitus.

**Figure 3:**
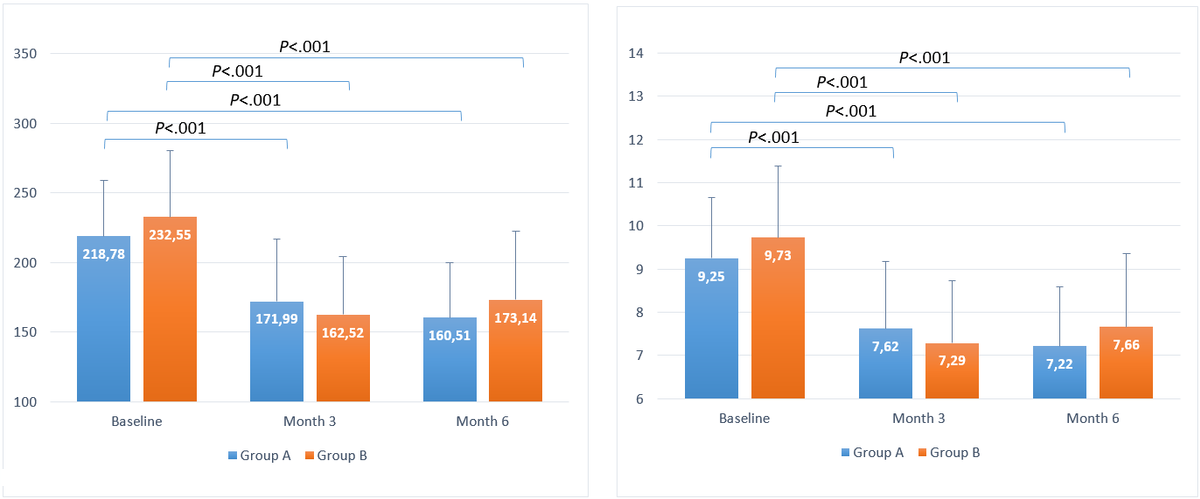
Timeline evolution of estimated blood glucose (left) and estimated glycated hemoglobin (right) for users with type 2 diabetes mellitus.

Baseline BG results for T1DM groups A and B were 213.61 (SD 31.57) mg/dL and 206.43 (SD 18.65) mg/dL, respectively, which decreased to 175.15 (SD 37.88) mg/dL and 180.60 (SD 40.47) mg/dL by month 6, respectively. The mean baseline BG reduction was 18% (*P*<.001) and 13% (*P*=.001) for groups A and B, respectively. For the T2DM groups, baseline BG level was 218.77 (SD 40.18) mg/dL and 232.55 (SD 46.78) mg/dL, respectively, which decreased to 160.51 (SD 39.32) mg/dL and 173.14 (SD 52.81) mg/dL by month 6 for groups A and B, respectively. The mean baseline BG reduction was 27% (*P*<.001) and 26% (*P*<.001) at month 6 for groups A and B, respectively. All statistical results are summarized in Table 1 and can be seen in Figures 2 and 3.

On the basis of BG reduction, this corresponds to a reduction of eA_1c_ of approximately 1.3% and 0.9% for T1DM groups A and B, respectively, and 2% for both T2DM groups A and B, respectively, and the statistical results are enumerated in  Table 2 and represented in Figures 2 and 3.

### Reduction of Hypoglycemia and Hyperglycemia Risk

T1DM baseline LBGI results for groups A and B were 5.2 (SD 3.9) and 4.4 (SD 2.3), respectively, which decreased to 3.4 (SD 3.3) and 3.4 (SD 1.9) by month 6, respectively; a reduction of 39% (*P*=.005) and 22% (*P*=.02), respectively, in the mean. The baseline HBGI results for groups A and B were 12.6 (SD 4.3) and 10.6 (SD 4.03), respectively, which decreased to 9.0 (SD 6.5) and 8.6 (SD 4.7) by month 6, respectively. The mean reduction in baseline HBGI was 30% (*P*=.001) and 22% (*P*=.003) for groups A and B, respectively.

For T2DM users, the baseline LBGI results for groups A and B were 1.52 (SD 1.15) and 2.62 (SD 1.76), respectively, which decreased to 1.13 (SD 1.14) and 2.12 (SD 0.96) by month 6, respectively. The mean reduction in baseline LBGI was 25% (*P*=.01) and 19% (*P*=.03) for groups A and B, respectively. For HBGI, the baseline results for groups A and B were 9.71 (SD 4.63) and 9.70 (SD 4.34), which decreased to 4.27 (SD 4.26) and 5.57 (SD 2.61) by month 6, respectively. The mean reduction in baseline HBGI was 56% (*P*<.001) and 44% (*P*<.001) for groups A and B, respectively.

**Table 1:** The evolution of blood glucose in type 1 diabetes and type 2 diabetes users.

Type and engagement		N	Blood glucose		
			Baseline, mean (SD)	Month 3, mean (SD)	Month 6, mean (SD)
**Type 1 diabetes**					
	High	82	213.61 (31.57)	177.45 (37.31)	175.15 (37.88)
	Low	62	206.42 (18.65)	179.46 (30.99)	180.60 (31.57)
**Type 2 diabetes**					
	High	45	218.78 (40.18)	171.99 (44.77)	160.51 (39.32)
	Low	22	232.55 (47.78)	162.52 (41.65)	173.14 (49.08)

**Table 2:** The evolution of estimated glycated hemoglobin in type 1 diabetes and type 2 diabetes users.

Type and engagement		N	Glycated hemoglobin		
			Baseline, mean (SD)	Month 3, mean (SD)	Month 6, mean (SD)
**Type 1 diabetes**					
	High	82	9.07 (1.10)	7.81 (1.30)	7.73 (1.32)
	Low	62	8.82 (0.65)	7.88 (1.08)	7.92 (1.10)
**Type 2 diabetes**					
	High	45	9.25 (1.40)	7.62 (1.56)	7.22 (1.37)
	Low	22	9.73 (1.66)	7.29 (1.45)	7.66 (1.71)

For T1DM users, the baseline LBGI results for groups A and B were 4.72 (SD 1.93) and 4.4 (SD 1.34), respectively, and decreased at t2 to 2.92 (SD 2.20) and 3.45 (SD 1), respectively; this was a reduction of 39% (*P*=.005) and 22% (*P*=.02), respectively, in the mean.

**Figure 4:**
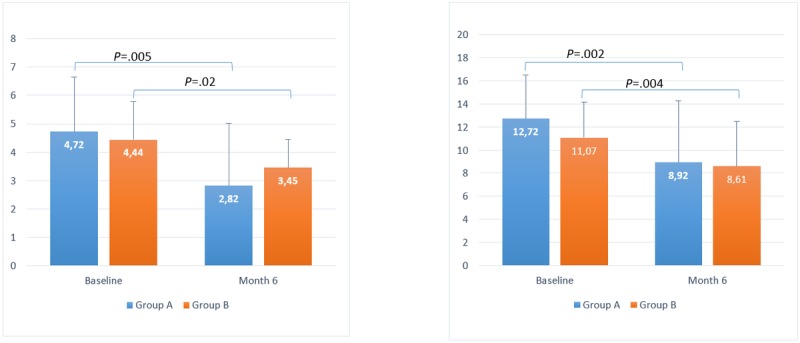
Timeline evolution of low blood glucose index (left) and high blood glucose index (right) for users with type 1 diabetes mellitus.

**Figure 5:**
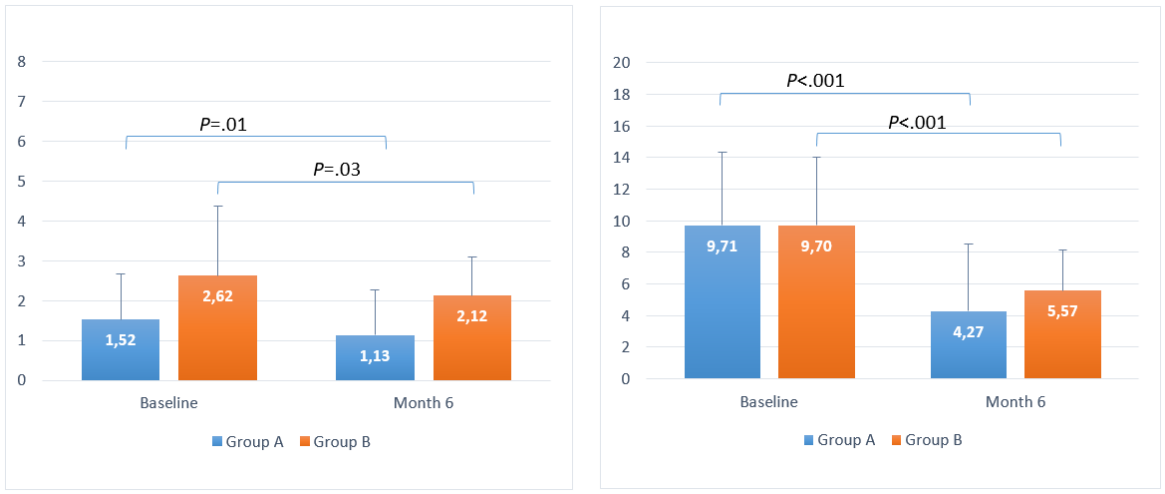
Timeline evolution of low blood glucose index (left) and high blood glucose index (right) for users with type 2 diabetes mellitus.

Baseline HBGI results for groups A and B were 12.72 (SD 3.79) and 11.07 (SD 3.07), respectively, and decreased at t2 to 8.92 (SD 5.35) and 8.61 (SD 3.86), respectively; this was a reduction of 30% (*P*=.002) and 22% (*P*=.004), respectively, in the mean. All statistical results are summarized in Table 3 and shown in Figures 4 and 5.

**Table 3:** The evolution of both low blood glucose index and high blood glucose index for type 1 diabetes and type 2 diabetes users.

Type and engagement		N	Low blood glucose index		High blood glucose index	
			Baseline, mean (SD)	Month 6, mean (SD)	Baseline, mean (SD)	Month 6, mean (SD)
**Type 1 diabetes**						
	High	486	4.44 (1.34)	3.45 (1.00)	9.73 (1.66)	8.61 (3.86)
	Low	76	4.72 (1.93)	2.92 (2.20)	12.72 (3.79)	8.92 (5.35)
**Type 2 diabetes**						
	High	73	2.62 (1.76)	2.12 (0.96)	9.70 (4.34)	5.57 (2.61)
	Low	38	1.52 (1.15)	1.13 (1.14)	9.71 (4.63)	4.27 (4.26)

## Discussion

### Principal Findings

Significant reductions in BG levels were found in all groups, independent of the use frequency of the app. We also found better outcomes for participants with T2DM.

Significant reductions in LBGI and HBGI were found in all groups, regardless of the use frequency of the app. LBGI and HBGI of both groups tended to have similar values after using the app for 6 months.

We also found a slight increase in the estimated HbA_1c_ between 3 and 6 months. However, as *P* value shows in all cases, this increment is not significant.

The positive change in both HbA_1c_ and risk indices for hypoglycemia and hyperglycemia, regardless of the frequency of use of the app by users, demonstrates the initial hypothesis, arguing that frequency of app use is not as important as the consistency and continuity of use. Thus, our hypothesis states that consistent and continuous app use results in an improvement of glycemic control, regardless of use frequency.

SDA has been shown to help patients by improving disease management, even during infrequent app use. Our guess is that this is because of the patient’s ability to learn disease management skills through the app, which aids in decision making and enables users to recognize specific situations, even without constant support from the app. Therefore, the higher-frequency use does not translate directly into improved performance or benefits, but rather an optimum between frequency and consistency is required.

### Limitations

In this study, we considered the limited number of participants and the access difficulty to the SDA database as study limitations. The main problem was the need of a project manager or an expert from the company to solve problems associated with the database to facilitate data reorganization. Therefore, there was a lack of communication between the app entity and our research group, which caused access to the SDA database to be difficult. In addition, because of this lack of reorganization, a significant amount of data was lost. For this reason, the number of participants was less than expected.

In more detail, 11,542 users were analyzed from more than 12,000 registered users in the SDA database with the accepted ethical consent. The problem was based on an error in the link that allowed users to save their controls. Sometimes, the link failed, and consequently, the controls were lost. A huge number of users were not able to register their own controls and could not be evaluated. Despite this, the number of users to be evaluated was enough because more than 95% of users could be evaluated.

The patients that have been analyzed in this study have been taken directly from the database and analyzed retrospectively. So, we do not have complete data on other sources of support/care/teaching that they may have had. However, in surveys conducted by the company in patient samples, no differences were detected between the different groups of patients.

### Conclusions

SDA is the first app that demonstrates that *the more use, the better* belief is not always optimal. In the case of SDA, the results are similar for both cases of lower-frequency and higher-frequency users. Using SDA may favorably impact glycemic control. Moreover, it is one of the few apps that may improve self-management for both T1DM and T2DM patients. As previously stated, the patient can learn how to manage his or her disease with this app, which increases patient empowerment and improves self-management, even for a very low use frequency.

Hence, we propose an innovative app, *SocialDiabetes*, as a self-management platform for people with any type of diabetes that aims to help people with diabetes live healthier, more comfortable lives. Through the activity of the patient, SDA provides tools, guidelines, and advice to the patient so that he/she can improve his/her management, knowledge, and self-care motivation toward his/her disease.

Pending studies to be carried out in the future include the study of the user’s experience and how this experience can improve patient empowerment. The improvement in the quality of life should also be studied.

## Abbreviations

BG: blood glucose
BMI: body mass index
DM: diabetes mellitus
eA_1c_: estimated A_1c_
HbA_1c_: glycated hemoglobin
HBGI: high blood glucose index
LBGI: low blood glucose index
mHealth: mobile health
SDA: SocialDiabetes app
T1DM: type 1 diabetes mellitus
T2DM: type 2 diabetes mellitus
